# Correlation between mammal track abundance and Forest Landscape Integrity Index validates actual forest ecological integrity

**DOI:** 10.1007/s00442-024-05613-z

**Published:** 2024-09-04

**Authors:** Francesca Malcangi, Andreas Lindén, Janne Sundell, John Loehr

**Affiliations:** 1 Lammi Biological Station, University of Helsinki, Pääjärventie 320, 16900 Lammi, Finland; 2 Natural Resources Institute Finland (Luke), 00790 Helsinki, Finland

**Keywords:** Ecological integrity, Habitat loss, Boreal forest, Mammals, Native species

## Abstract

Human disturbance compromises the ecological integrity of forests, negatively affecting associated species. Assessing the impact of forest integrity on biodiversity is complex due to the interplay of various human activities, ecological factors, and their interactions. Current large-scale indices assess forest integrity but often lack a direct connection to the biotic environment. We tested the effectiveness of the global Forest Landscape Integrity Index (FLII) in evaluating aspects of anthropogenic forest degradation on the biotic community. We analyzed the relationship between changes in the ecological integrity of Finnish forests and variations in mammal species abundance, using the number of tracks from 17 different species collected during the winter seasons between 2016 and 2020 in south-central Finland. Beyond the FLII, we analyzed forest and canopy cover to enhance the accuracy of habitat preference assessments. We found that the FLII captures the varying degrees of forest integrity, as reflected by the correlation between the abundance of winter tracks and the FLII for most mammals. Species that were positively associated with forest integrity were all native to the boreal forest, while mammals that adapt well to human-disturbed environments including two invasive species were more common in lower FLII forests. Significant differences in habitat preferences were also observed in relation to forest and canopy cover, revealing additional nuances that the FLII alone did not capture. This study demonstrates that the FLII, when combined with a comprehensive dataset and supplemented with region-specific factors, can assess species' adaptability to human-modified forests, aiding in the development of conservation strategies.

**Supplementary Information:**

The online version contains supplementary material available at 10.1007/s00442-024-05613-z.

## Introduction

The degradation of natural habitats due to human activities is the major driver of biodiversity loss globally (Storch et al. 2022). In forests worldwide, tree cutting for logging and other land uses, such as agriculture, livestock areas, and urbanization, are primary causes of disturbance (Seidl and Turner 2022). These activities alter the ecological integrity of forests, defined as the degree to which an ecosystem remains free from anthropogenic changes in its structure, composition, and function, leading to negative consequences for many associated species (Parrish et al. 2003; Grantham et al. 2020).

Forest disturbances, both natural and human-induced, have significant ecological consequences. In boreal forests, for instance, natural events, such as fires and insect outbreaks, can cause substantial impacts. In addition to natural disturbances human activities, particularly those that are intense and prolonged, add further pressure on ecosystems (Rockström et al. 2009). These disturbances can lead to changes in community composition (Hagen et al. 2012; Venier et al. 2014) and ecosystem functionality (Cardinale et al. 2006; Folke et al. 2004), ultimately resulting in the loss of essential ecosystem services with significant socioeconomic impacts (Cardinale et al. 2012).

In Finland, despite having the highest forest cover in Europe—over 75% according to the Ministry of Agriculture and Forestry—42% of all species in Finland live in these forests, and 30% of the threatened species use them as their primary habitat. The Red List 2019 indicates that of the 58 mammal species assessed, around 21% are classified as threatened (Hyvärinen et al. 2019).

Critical factors leading to species endangerment or extinction include changes in forest habitats due to forest management activities, the reduction of old-growth forests, large trees, and the decreasing amounts of dead and decaying wood (Jonsson et al. 2016; Mönkkönen et al. 2022). The challenge of achieving sustainable forest use in Finland arises from the intensive industrial exploitation of boreal forests, primarily through systematic silvicultural practices. This excessive use severely damages ecologically essential forest structures and processes, reducing habitat quality and the ecological capacity to support various native species vulnerable to environmental disturbances, thereby altering the composition of forest-dependent species communities (Semenchuk et al. 2022).

Given these impacts, it is crucial to consider the potential effects of landscape changes on species abundance and distribution, and to implement measures to mitigate any negative consequences. However, making informed decisions on biodiversity is challenging for policymakers due to the lack of clear, measurable targets and the complexity of ecosystems, which complicates the assessment for the public and stakeholders (Oettel et al. 2021).

Several studies have highlighted the need for indices that integrate both abiotic and biotic data to more accurately reflect ecological integrity (Weigel et al. 2002; Zhao et al. 2019; Zelený et al. 2021). Despite this, assessing forest integrity and degradation at a global scale is complex and requires reliable proxies, which often do not have a direct connection to the specific biotic environment (Pan et al. 2021; Jia et al. 2023).

The first index to assess the ecological integrity of forests on a global scale is the Forest Landscape Integrity Index (FLII). It was developed to provide a comprehensive measure of forest ecological integrity by combining observed reductions in forest connectivity with proxy data on human activity intensity known to cause forest degradation (Grantham et al. 2020). The FLII has revealed important insights into the state of the world's forests, showing, for example, that boreal forests in northern Russia, Canada, and Alaska have large areas of high integrity, whereas regions like Fennoscandia, including Finland, show low overall ecological integrity. In Finland, the national average FLII value is 5.08 out of a maximum of 10, with particularly low scores in southern Finland. This low ecological integrity can be correlated with a higher concentration of threatened and red-listed species in these areas, highlighting the strong pressure exerted on their habitats by various land-use forms (Hyvärinen et al. 2019).

In this context, understanding whether the FLII represents the ecological condition of the forest, such as the diversity and abundance of the species that inhabit it, can provide valuable insights into the relationship between forest integrity and species distribution, aiding in the development of conservation strategies for increasingly anthropized areas.

The aim of this study is to assess whether the Forest Landscape Integrity Index (FLII) is a robust proxy for measuring forest integrity in relation to the biotic conditions, particularly in the context of mammal abundance differences. This study also explores the potential of forest and canopy cover variations, which are not taken into account by FLII. This helps us to better investigate which habitat characteristics are important for the survival and thriving of different animal species in human-modified forests. By doing so, we can improve our understanding of the effective FLII’s application at fine-scale and offer valuable insights to address the challenge of evaluating the connection between forest integrity degradation and species status.

## Materials and methods

### Wildlife triangles

The data on wild mammal abundance were based on the wildlife triangle scheme, which is a monitoring scheme of wildlife abundance organized by the Natural Resources Institute Finland (Pellikka et al. 2005). The scheme is based on a network of triangle-shaped 4 × 4 × 4 km transects (totaling 12 km per triangle), with fixed locations covering the entire country. The spatial location of each triangle was available as the center point of the triangle. Mammal snow tracks have been surveyed each winter since 1989, at approximately 700 triangles located randomly in forested areas throughout Finland and the survey period is between January 15th and February 28th in southern and central Finland.

All tracks of 24 mammal species crossing the transect are recorded. Counts are performed one or a few days after a snowfall or pre-count. During the pre-count, old tracks are marked to distinguish them from new tracks that accumulate afterward. Considering the distance surveyed and the number of days since the last snowfall, a snow track index representing the number of snow tracks per 10 km per day is calculated.

In addition to the tracks counted mainly in forested landscapes, triangles in agricultural areas were also included in the analyses. These so-called “Field triangles” have sides of 2 km, totaling 6 km of tracking route, and are situated in mosaic-like landscapes where approximately half of the census lines traverse farmland, while the remainder passes through forests, yards, and built-up areas. Since their establishment in 1999, field triangles have been regularly censused, primarily located in Western and Southern Finland, where agricultural activity is most prevalent.

**Fig. 1:**
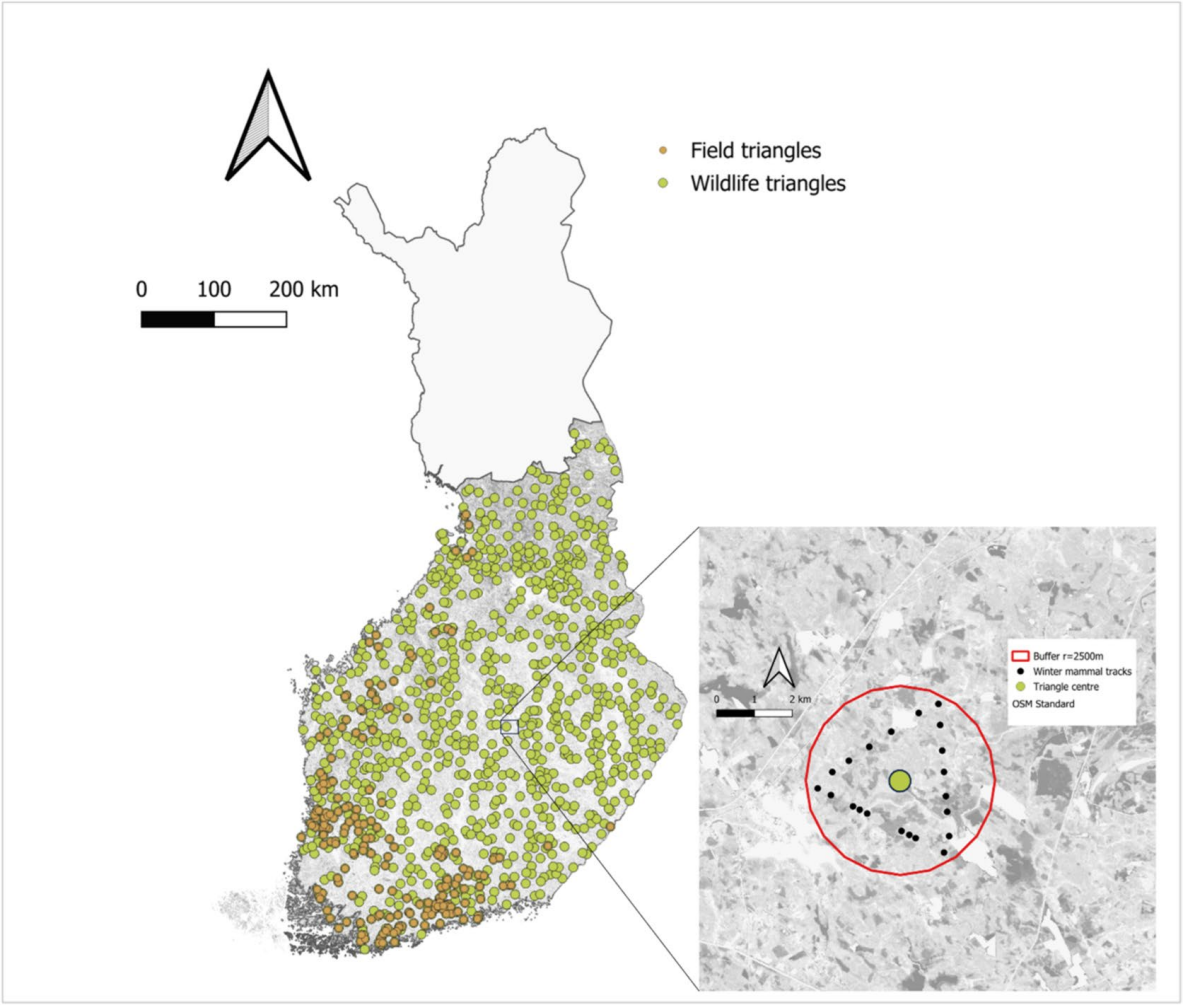
Track data collected from wildlife (in green) and field (in orange) triangles. At the bottom right of the figure is an example of a 2.5 km buffer around the center of a triangle with the tracks of different mammal species (in black)

Our study utilized snow track data collected from both wildlife and field triangles (Fig. 1), totaling 1063 triangle years distributed across 2016 to 2020 (247 triangles sampled in 2016, 217 in 2017, 230 in 2018, 182 in 2019, and 187 in 2020).

### Study species

The analysis focused on 17 of the 24 mammal species surveyed in the winter wildlife triangle counts. The species were selected based on their relative abundance in the available data, with those having fewer than 100 observations in total in the study area being excluded (The species and their relative abundance are reported in the Supplementary tables S1 and S2).

We analyzed the following species that are native to the boreal forest: the mountain hare (Lepus *timidus, L.*), moose (*Alces alces, L.*), forest reindeer (*Rangifer tarandus fennicus, L.*), wolverine (*Gulo gulo, L.*), Eurasian lynx (*Lynx lynx, L.*), wolf (*Canis lupus, L.*), pine marten (*Martes martes, L.*), least weasel (*Mustela nivalis nivalis,L.*), stoat (*Mustela erminea, L.*), Eurasian otter (*Lutra lutra*), red squirrel (*Sciurus vulgaris, L.*), and red fox (*Vulpes vulpes, L.*). Although many of these species have distributions that include habitats other than the Eurasian taiga biome, they all are native to this environment. Some of them benefit from coexistence with humans in terms of increased density, reproduction, and/or survival. Among these, the red fox is considered a generalist species (Wallén et al. 2018). The observed increase in fox populations can be attributed to human activities, which provide red foxes with more scavenging opportunities, habitats for preferred prey (e.g., voles), and higher predation success by increasing the amount of habitat edges (Jahren et al. 2020; Gorini et al. 2012; Güthlin et al. 2013; Vuorisalo et al. 2014). Red squirrel populations in Finland are more abundant in urban and rural areas than in forests. One factor that could explain this phenomenon is the increase in winter feeding sites in urban environments, especially during the harvest period of spruce cones, which are the main food of red squirrels (Jokimäki et al. 2017). Finally, the mustelids include the least weasel and stoat, which prefer farmland habitats, especially during the peak density phase of the *Microtus* vole on which they feed (Klemola et al. 1999). Other studies have found that fragmented agricultural landscapes with residual edge habitats favor the cover and food requirements of these species (Magrini et al. 2009).

Our analysis also included invasive species present in the study area: the brown hare (*Lepus europaeus, P.*) and roe deer (*Capreolus capreolus, L.*), which naturally dispersed into Finland and have become abundant only in the last 50–150 years, showing a high ability to tolerate anthropogenic land use and habitat modifications (Levänen et al. 2019a, b; Linnell et al. 2020; Tasser et al. 2023). White-tailed deer (*Odocoileus virginianus, L.*), raccoon dog (*Nyctereutes procyonoides, L.*), and American mink (*Neogale vison*), which were introduced by humans and whose populations were successful and increased rapidly in the new areas (Kauhala and Helle 1995; Bonesi and Palazon 2006; Poutanen et al. 2023).

### Study area

We included data from the southern and central parts of Finland in our analyses (Fig. 1). The study area also encompasses the wildlife districts and associations known as 'riistakeskus' and 'rhy,' where the survey triangles are geographically grouped. The main reason we restricted our analysis to this study area is that all species are found there. However, for some species with a narrower distribution range—in particular the forest reindeer, lynx, wolverine, wolf, roe deer, white-tailed deer, brown hare and raccoon dog—we restricted our analysis to geographical areas with a relevant number of track observations, constructing a minimum convex polygon (MCP) or convex hull of all positive observations in the data for the years considered. By excluding Northern Finland, it was also possible to separate the effects of latitude and forest integrity without encountering multicollinearity problems with these variables.

### Explanatory variables

Initially, we tested whether FLII is a good proxy for forest ecological integrity by analyzing its association with the abundance of species tracks. Subsequently, in addition to FLII, we also analyzed forest cover and canopy cover to gain a more complete understanding of habitat suitability for mammal species. This is because, although the FLII provides an assessment of anthropogenic disturbances in the forest, changes in forest structure, which can be an important indicator of species' forest preferences, forest cover and canopy cover are not directly extractable from the FLII score. In particular, the inclusion of these two variables makes it possible to distinguish areas that have the same level of anthropogenic disturbance (as indicated by the FLII), but differ significantly in their physical structure and, consequently, in their suitability as habitat for different mammal species.

#### Forest Landscape Integrity Index (FLII)

The FLII is the first tool designed to infer the ecological integrity of forests for conservation, management, and restoration at global scale. It is constructed based on three main aspects: 1) observed pressures, 2) inferred pressures, and 3) changes in forest connectivity. The definition of forest in the FLII baseline dataset includes a 20% canopy cover threshold and all woody vegetation greater than 5 m in height, encompassing naturally regenerated forests, tree crops, planted forests, wooded agroforests, and urban tree cover.

The observed pressures include direct human impacts, such as infrastructure, agriculture, and deforestation. These pressures are quantified using spatial datasets that map the presence and intensity of these activities. Each type of pressure is assigned a weight based on its estimated impact on forest integrity. The weighted values are then combined to form a composite measure of observed pressures for each forest pixel, ensuring that areas with higher levels of human activity are appropriately reflected in the index.

For the anthropogenic pressures that were inferred, Grantham et al. (2020) accounted for indirect impacts, such as the creation of microclimates, species interactions at forest margins, hunting, firewood collection, pollution, livestock grazing, and fire spread. The impacts were modeled based on their spatial association with observed pressures, with intensity declining with distance.

Changes in forest connectivity were determined by comparing the current forest configuration with a potential configuration that assumes no extensive human alterations. This comparison indicates the degree of forest fragmentation. These values were spatially applied to a global forest extent map for early 2019 by subtracting the 2001–2018 annual tree cover loss from the Global Tree Cover 2000 product. The final base map for 2019 was generated from 300 m resolution pixels classified as forest or non-forest according to a majority rule. Finally, these elements were combined to generate an FLII score for each forest pixel, reflecting overall forest integrity. Higher scores indicate less human impact and better ecological integrity.

The FLII was calculated for each forest pixel, but not for non-forest pixels. We downloaded the FLII map used in this study from the official website (https://www.forestintegrity.com/) in tiff format for Europe with a WGS 1984/300 resolution per pixel and the data multiplied by 1000 to store them in full format.

#### Canopy cover

The FLII described earlier uses a minimum threshold of 20% canopy cover to define a forest; however, this threshold represents a simple binary classification to distinguish between forest and non-forest and does not consider more detailed information about the varying amounts of canopy cover. To consider this we used geographic data on canopy cover (%), estimated in 2019, defined as the percentage of forest floor covered by the vertical projection of tree crowns. We obtained these data from the multiple source inventory operating system, which uses field inventories, satellite images, numerical base maps, and contour maps (Tomppo et al., 1998; Ministry of Agriculture and Forestry, 2000). The maps are in raster format with a pixel size of 16 × 16 m and in the ETRS-TM35FIN coordinate system. Cover values were obtained by Korhonen et al. (2007) through a modeling technique, beta regression, specifically designed to model percentage variables such as canopy cover. For forest soils and poorly productive soils, estimation was done separately based on dominant species Scots pine (*Pinus sylvestris L*.), Norway spruce (*Picea abies L*.), and others. Deciduous tree canopy cover was calculated based on the proportion of deciduous trees in the plot (calculated from stem counts in seedling stands and basal area of immature stands).

#### Forest cover

To account for the proportion of forest that characterizes the study area versus the percentage of area characterized by other land-use categories, we also analyzed forest cover, described as the amount of forest that covers a particular area of land. In our case, we obtained forest cover by calculating from the canopy cover raster the percentage ratio between the number of pixels containing forest and the number of pixels with no values for the canopy cover variable. This measure helps distinguish between areas with similar FLII scores but varying forest density and configuration. In particular, whereas forest cover provides a quantitative measure of the physical space occupied by forests, which can directly influence the habitat available for species, FLII adds a qualitative dimension by assessing the ecological integrity of these forests.

### Spatial analysis

Geospatial analysis characterized the first stage of evaluating the relationship between the explanatory variables and the number of tracks recorded for each species in the wildlife triangles (Fig. 1). We calculated the average FLII and the percentage of other covariates within a buffer with a radius of 2500 m around the georeferenced center of each triangle. This approach ensured that we captured the relevant habitat characteristics influencing species presence. We selected tracks of one species at a time and accounted for the geographic distribution of each individual species by including only triangles within the geographic area where tracks of a given species were found to be present. We used QGIS version 3.32 to perform this analysis.

### Statistical analysis

We modeled the relationship between the snow track index (the response variable) for each species and the explanatory variables described above using a generalized linear mixed model (GLMM) with a logarithmic link function and a negative binomial error distribution. The number of tracks observed in a triangle for the species under study was technically the response variable. However, we modeled the snow track index (tracks/10 km/day) by including an offset variable—the natural logarithm of the survey effort (Lindén and Piha 2024). The survey effort was represented by the distance surveyed, which is typically around 12 km (1.2 units of 10 km), although this distance can vary slightly due to practical surveying conditions. Additionally, we included the logarithm of days since the last snowfall as a covariate. This allowed us to account for changes in the number of tracks observed with the days since the last snowfall. We only considered days since the last snowfall less than or equal to five to account for the nonlinearity between the number of tracks and days since the last snowfall.

The ID of the wildlife triangle was included in the model as a random effect on the intercept to account for the non-independence due to the same triangle being surveyed in different years. We also considered spatial autocorrelation between triangles by adding the wildlife districts ("riistakeskus") and wildlife associations ("rhy"), where the triangles are geographically grouped, thus defining a nested random effect in our models.

In the first model, called 'FLII model,' we analyzed FLII and the latitude of the triangle location as the main explanatory variables (fixed effects). Latitude was included as a control variable to account for potential climatic and environmental gradients that may influence species abundance, ensuring that the effects of forest-related variables are not confounded by latitudinal variations. In the second model, called 'full model,' we included forest cover and canopy cover in addition to FLII and latitude. For all covariates, we applied centering, a technique in which the mean of the independent variables is subtracted from all values (Schielzeth 2010). This ensures that all independent variables have a mean of zero, helping the model to work efficiently. The models were fitted using the "glmmTMB" package (Brooks M.E. et al. 2017) in the R programming environment version 4.2.1 (R Core Team, 2022). In each model, those with *p*-value < 0.05 and 95% confidence intervals of the estimated effects not including zero were considered significant.

To evaluate the effectiveness of the Forest Landscape Integrity Index (FLII) in predicting mammal abundance, we calculated the coefficient of determination R^2^ using a method adapted from Nakagawa and Schielzeth (2013) for generalized linear mixed-effects models. This method allows us to calculate both marginal R^2^ (variance explained by fixed effects) and conditional R^2^ (variance explained by both fixed and random effects). The conditional R^2^ also includes distribution-specific variance and residual variance due to overdispersion.

When calculating the marginal R^2^, we excluded offset and days since last snowfall, as these are effort variables used to account for the structure of the snow track index in our models.

We also calculated the standardized coefficients of the fixed effects explanatory variables to illustrate their effect sizes, and predictive abilities in relation to each other (Schielzeth 2010). The standardized coefficients and their standard errors (SE) were calculated by multiplying the original estimates with the sample standard deviations of the explanatory variables. Hence, we standardize only with respect to the explanatory variables (Newman and Browner 1991), not all input variables, as is typically applied for linear regressions.

We assessed potential multicollinearity among the predictor variables using Variance Inflation Factor (VIF) analysis. In general, the VIF values were low for all variables, indicating low multicollinearity. Specifically, the VIF values for FLII ranged from 1.21 to 2.56, while those for forest cover ranged from 1.08 to 1.56. These values suggest low multicollinearity among these predictors (Zuur et al. 2009).

Additionally, residual diagnostics were checked using the ‘DHARMa’ package (Hartig and Lohse 2022). The ‘DHARMa’ package uses a simulation-based approach to create readily interpretable scaled (quantile) residuals for fitted (generalized) linear mixed models. The resulting residuals are standardized to values between 0 and 1 and can be interpreted as intuitively as residuals from a linear regression.

## Results

**Fig. 2:**
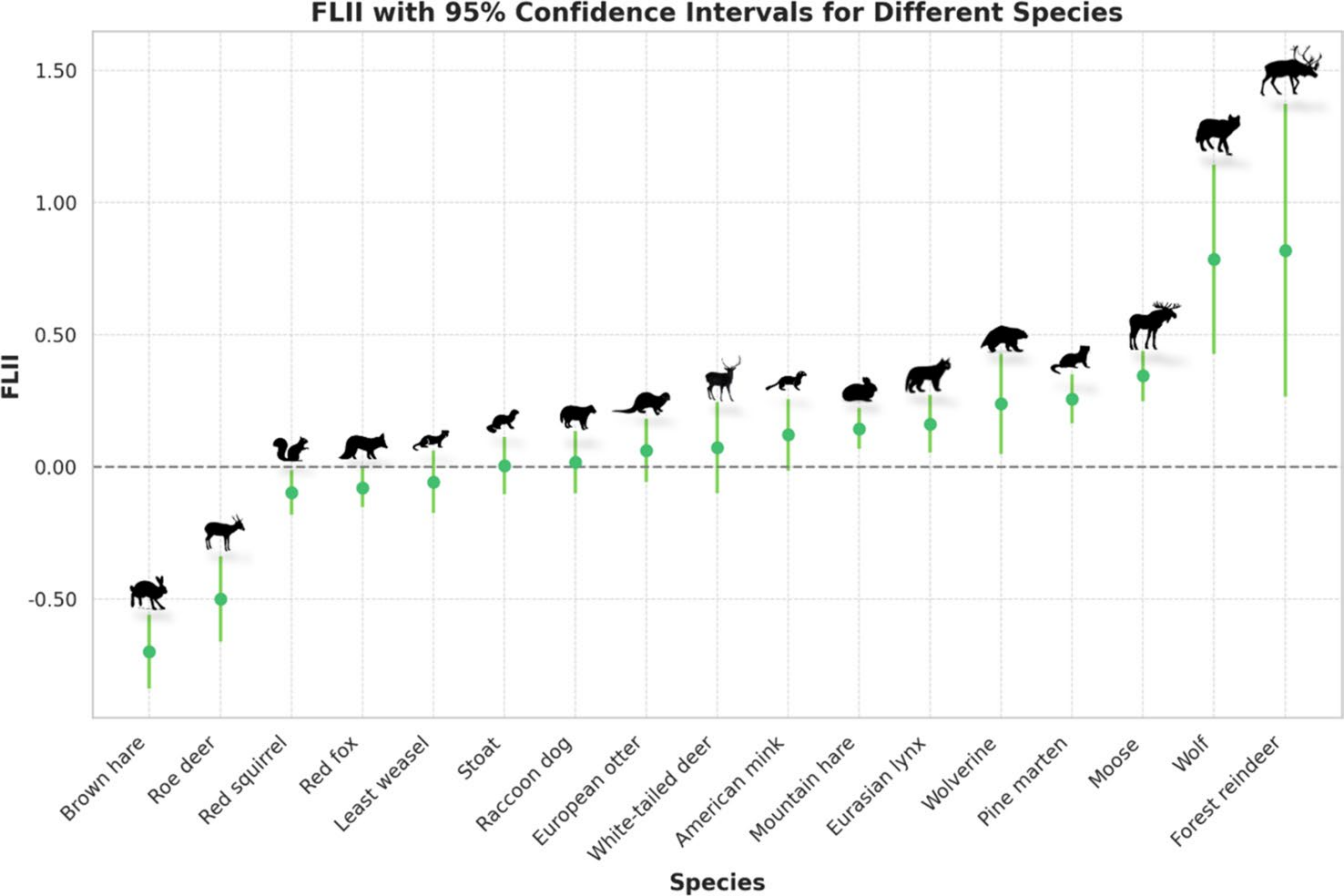
The error bar plot illustrates the FLII estimated coefficients values and the relative 95% confidence intervals for each species obtained from the FLII model

We analyzed the relationship between the Forest Landscape Integrity Index (FLII) and snow tracks for 17 different species (Table 1, Fig. 2). We found that the FLII was positively associated with the abundance of seven of the studied species, including the Eurasian lynx, forest reindeer, moose, mountain hare, pine marten, wolf, and wolverine. A negative association was found for the brown hare, roe deer, red fox, and red squirrel. Observing the multiplicative effects of FLII on abundance, measured as standardized coefficients (beta), we found very large positive effects for the forest reindeer and wolf (beta = 1.3–1.4), and large negative effects for the brown hare and roe deer (beta = − 0.8 to − 1.2). Additionally, high positive effects were observed for the moose, pine marten, and wolverine (beta = 0.4–0.6), and moderately high positive effects for the Eurasian lynx and mountain hare (beta = 0.24–0.28) (Supplementary Table S3).

**Table 1:** Parameter estimates from species-specific GLMMs examining factors affecting snow track abundance in wildlife triangle censuses (2016–2020)

Species	Intercept	FLII	Latitude	Districts variance	Associations variance	Triangles variance	Dispersion parameter	R^2^m	R^2^c	Triangles sampled
American mink	**− 2.160 (**− **2.577 **− **1.743)**	0.121 (− 0.015 0.256)	**0.279 (0.072 0.485)**	0.205	0.117	2.151	0.723	0.074	0.517	1063
Brown hare	− 0.394 (− 0.996 0.207)	**− 0.700 (− 0.839 − 0.561)**	**− 0.641 (− 0.974 − 0.307)**	1.026	0.589	3.421	1.475	0.348	0.896	871
Eurasian lynx	**− 0.697 (− 1.183 − 0.210)**	**0.162 (0.053 0.271)**	− 0.213 (− 0.455 0.028)	0.611	0.271	1.270	0.659	0.018	0.454	995
European otter	**− 1.835 (− 2.139 − 1.531)**	0.061 (− 0.059 0.182)	− 0.012 (− 0.197 0.173)	0.020	0.142	2.219	1.29	0.02	0.681	1063
Forest reindeer	**− 3.465 (− 5.420 − 1.511)**	**0.820 (0.266 1.375)**	− 0.139 (− 1.178 0.900)	3.637	3.812	1.44E-07	0.033	0.002	0.01	568
Least weasel	**− 0.838 (− 1.089 − 0.586)**	− 0.057 (− 0.176 0.062)	− 0.018 (− 0.146 0.110)	8.66E-09	0.041	2.774	0.665	0.011	0.483	1063
Moose	**1.776 (1.549 2.002)**	**0.344 (0.248 0.439)**	**− 0.263 (− 0.403 − 0.122)**	0.085	0.180	2.348	1.365	0.051	0.726	1063
Mountain hare	**3.062 (2.791 3.332)**	**0.144 (0.068 0.221)**	0.054 (− 0.097 0.205)	0.195	0.091	1.752	2.311	0.044	0.8	1063
Pine marten	0.082 (− 0.229 0.392)	**0.256 (0.163 0.350)**	− 0.065 (− 0.231 0.102)	0.207	0.186	1.729	1.079	0.041	0.607	1063
Raccoon dog	**− 1.477 (− 1.869 − 1.085)**	0.018 (− 0.100 0.135)	**− 0.952 (− 1.149 − 0.755)**	0.171	0.341	1.373	0.406	0.213	0.378	1005
Red fox	**1.821 (1.517 2.126)**	**− 0.079 (− 0.154 − 0.003)**	− 0.133 (− 0.296 0.030)	0.247	0.319	1.492	3.013	0.045	0.847	1063
Red squirrel	**0.969 (0.817 1.121)**	**− 0.098 (− 0.183 − 0.013)**	**− 0.221 (− 0.316 − 0.125)**	8.05E-09	0.139	1.816	1.352	0.082	0.674	1063
Roe deer	**− 1.759 (− 2.564 − 0.954)**	**− 0.501 (− 0.662 − 0.339)**	**− 0.631 (− 1.029 − 0.232)**	1.828	1.013	4.194	0.768	0.217	0.798	944
Stoat	**− 0.459 (− 0.680 − 0.237)**	0.004 (− 0.105 0.113)	**0.367 (0.254 0.481)**	1.40E-07	0.007	1.980	0.675	0.084	0.449	1063
White-tailed deer	− 0.789 (− 1.878 0.301)	0.072 (− 0.101 0.245)	**− 2.260 (− 2.836 − 1.683)**	3.395	0.839	3.606	1.132	0.392	0.909	683
Wolf	**− 4.128 (− 5.530 − 2.727)**	**0.786 (0.428 1.145)**	− 0.271 (− 0.801 0.259)	0.374	3.638	0.732	0.061	0.008	0.025	861
Wolverine	**− 3.371 (− 4.168 − 2.573)**	**0.238 (0.048 0.427)**	**0.943 (0.481 1.405)**	0.772	1.690	2.200	0.674	0.228	0.697	678

The table includes coefficients for FLII and latitude, their 95% confidence intervals, and significant values in bold. Nested random effect variances and model dispersion parameter are also presented, along with marginal and conditional R^2^ values indicating variance explained by the fixed effects and the total model, respectively

Significant coefficients, with p-values < 0.05 and 95% confidence intervals not including zero, were highlighted in bold

**Fig. 3:**
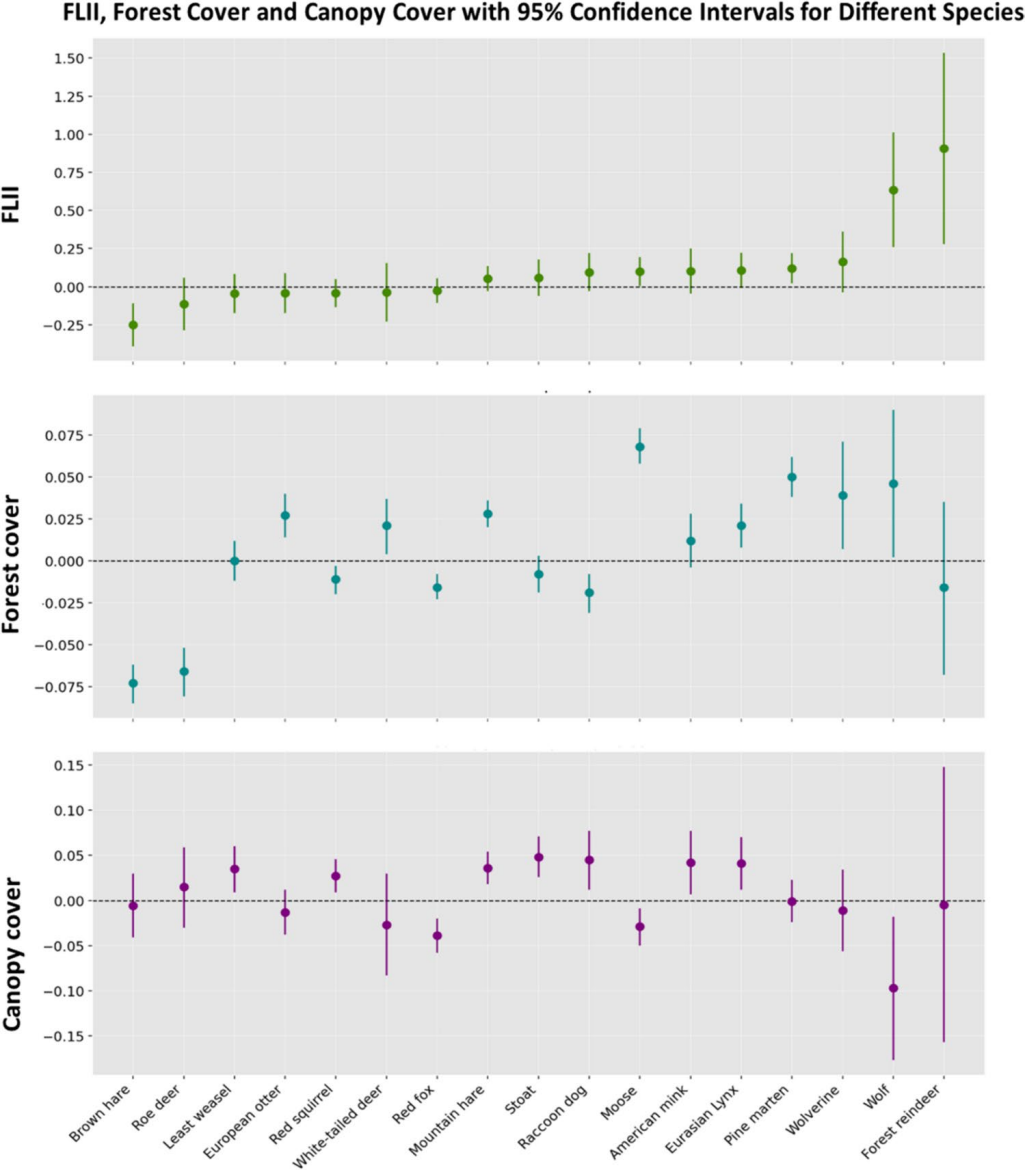
Error bars plot illustrates the estimated coefficients values and the relative 95% confidence intervals of FLII, forest cover and canopy cover for each species obtained from the full model

We then tested the full model, which included forest cover and canopy cover as additional independent variables along with FLII (Table 2, Fig. 3). Our results suggest that forest cover was positively associated with the abundance of the same species that showed a positive correlation with FLII in the FLII model, and in addition for the white-tailed deer and European otter. In contrast, for other species, particularly the brown hare, raccoon dog, roe deer, red fox, and red squirrel, we observed an increase in track abundance with decreasing forest cover. Additionally, our analysis revealed that canopy cover was positively correlated with snow track abundance for the American mink, Eurasian lynx, least weasel, mountain hare, raccoon dog, red squirrel and stoat. Conversely, for the moose, red fox, and roe deer and wolf, we found a negative relationship.

**Table 2:** Parameter estimates from species-specific GLMMs examining factors affecting snow track abundance in wildlife triangle censuses (2016–2020)

Species	Intercept	FLII	Forest cover	Canopy cover	Latitude	Districts variance	Associations variance	Triangles variance	Dispersion parameter	R^2^m	R^2^c
American mink	**− 2.212 (− 2.592 − 1.832)**	0.103 (− 0.044 0.250)	0.012 (− 0.004 0.028)	**0.042 (0.007 0.077)**	**0.359 (0.159 0.558)**	0.102	0.027	2.402	0.765	0.100	0.556
Brown hare	− 0.268 (− 0.665 0.129)	**− 0.250 (− 0.391 − 0.108)**	**− 0.073 (− 0.085 − 0.062)**	− 0.006 (− 0.041 0.030)	**− 0.573 (− 0.822 − 0.324)**	0.353	0.320	2.810	1.475	0.442	0.880
Eurasian Lynx	**− 0.727 (− 1.171 − 0.284)**	0.107 (− 0.009 0.224)	**0.021 (0.008 0.034)**	**0.041 (0.012 0.070)**	− 0.170 (− 0.406 0.066)	0.466	0.290	1.618	0.668	0.043	0.461
European otter	**− 1.889 (− 2.143 − 1.636)**	− 0.042 (− 0.172 0.089)	**0.027 (0.014 0.040)**	− 0.013 (− 0.038 0.012)	− 0.089 (− 0.227 0.050)	1.10E-10	3.88E-07	2.475	1.417	0.044	0.723
Forest reindeer	**− 3.435 (− 5.423 − 1.448)**	**0.907 (0.280 1.534)**	− 0.016 (− 0.068 0.035)	− 0.005 (− 0.157 0.148)	− 0.145 (− 1.213 0.924)	3.467	3.766	1.27E-07	0.033	0.002	0.010
Least weasel	**− 0.848 (− 1.095 − 0.600)**	− 0.044 (− 0.174 0.085)	0.000 (− 0.012 0.012)	**0.035 (0.009 0.060)**	0.042 (− 0.093 0.178)	2.89E-13	1.82E-07	2.831	0.670	0.018	0.492
Moose	**1.734 (1.523 1.944)**	**0.099 (0.003 0.195)**	**0.068 (0.058 0.079)**	**− 0.029 (− 0.050 − 0.009)**	**− 0.415 (− 0.551 − 0.279)**	0.071	0.110	2.044	1.370	0.202	0.743
Mountain hare	**3.068 (2.903 3.232)**	0.053 (− 0.029 0.135)	**0.028 (0.020 0.036)**	**0.036 (0.018 0.054)**	**0.118 (0.005 0.231)**	0.045	0.054	1.724	2.313	0.124	0.800
Pine marten	0.023 (− 0.208 0.255)	**0.121 (0.023 0.219)**	**0.050 (0.038 0.062)**	− 0.001 (− 0.024 0.023)	**− 0.163 (− 0.305 − 0.021)**	0.066	0.150	1.704	1.092	0.139	0.630
Raccoon dog	**− 1.474 (− 1.869 − 1.079)**	0.095 (− 0.030 0.219)	**− 0.019 (− 0.031 − 0.008)**	**0.045 (0.012 0.077)**	**− 0.853 (− 1.055 − 0.650)**	0.181	0.344	1.236	0.399	0.226	0.377
Red fox	**1.830 (1.557 2.103)**	− 0.026 (− 0.107 0.055)	**− 0.016 (− 0.023 − 0.008)**	**− 0.039 (− 0.058 − 0.020)**	**− 0.189 (− 0.343 − 0.035)**	0.189	0.264	1.464	3.011	0.083	0.844
Red squirrel	**0.965 (0.813 1.117)**	− 0.042 (− 0.134 0.050)	**− 0.011 (− 0.020 − 0.003)**	**0.027 (0.009 0.046)**	**− 0.161 (− 0.262 − 0.060)**	1.56E-08	0.141	1.785	1.351	0.097	0.676
Roe deer	**− 1.669 (− 2.350 − 0.988)**	− 0.114 (− 0.286 0.058)	**− 0.066 (− 0.081 − 0.052)**	0.015 (− 0.030 0.059)	**− 0.521 (− 0.875 − 0.166)**	1.219	0.754	3.713	0.762	0.257	0.774
Stoat	**− 0.468 (− 0.690 − 0.246)**	0.059 (− 0.059 0.178)	− 0.008 (− 0.019 0.003)	**0.048 (0.026 0.071)**	**0.468 (0.344 0.592)**	2.42E-09	0.024	1.927	0.680	0.098	0.456
White-tailed deer	− 0.792 (− 1.897 0.312)	− 0.037 (− 0.231 0.157)	**0.021 (0.004 0.037)**	− 0.027 (− 0.083 0.030)	**− 2.321 (− 2.903 − 1.739)**	3.497	0.868	3.535	1.132	0.394	0.910
Wolf	**− 4.174 (− 5.448 − 2.899)**	**0.635 (0.258 1.013)**	**0.046 (0.002 0.090)**	**− 0.097 (− 0.177 − 0.018)**	− 0.492 (− 1.042 0.057)	0.289	3.827	0.430	0.059	0.010	0.025
Wolverine	**− 3.409 (− 4.225 − 2.594)**	0.163 (− 0.037 0.362)	**0.039 (0.007 0.071)**	− 0.011 (− 0.056 0.034)	**0.877 (0.391 1.362)**	0.836	1.604	2.223	0.677	0.245	0.705

The table includes coefficients for FLII, latitude, forest cover, and canopy, their 95% confidence intervals, and significant values in bold. Nested random effect variances and model dispersion parameter are also presented, along with marginal and conditional R^2^ values indicating variance explained by the fixed effects and the total model, respectively

Significant coefficients, with p-values < 0.05 and 95% confidence intervals not including zero, were highlighted in bold

We calculated the marginal and conditional R^2^ values for both the FLII model (Table 1) and the full model, including forest cover and canopy cover as independent variables (Table 2). The results indicate that the full model explains more variance overall than the FLII model. Specifically, the marginal R^2^ values showed significant variation among different species. Higher marginal R^2^ values (> 0.3) model were found for the brown hare and white-tailed deer, indicating that the fixed effects in the model explain a considerable portion of the variance in the snow track index for these species. In contrast, species like the forest reindeer and the wolf had very low marginal R^2^ values (< 0.01), indicating that the FLII model explains only a small fraction of the variance for these species.

The inclusion of forest and canopy cover variables significantly improved the marginal R^2^ values for several species. This improvement was particularly pronounced, with an increase of at least 5%, for the brown hare, moose, mountain hare, and pine marten. Notably, for the moose and mountain hare, all the added variables were statistically significant. However, for certain species, such as the forest reindeer, the increase was marginal, indicating that these additional factors do not substantially enhance the explanation of variance for these species.

According to the effect sizes of the explanatory variables (Supplementary Table S4), we observed that in the full model forest cover had a predominant effect, decreasing the explanatory power of FLII for most species. Although canopy cover also contributed to the full model, its importance appeared less significant than forest cover.

## Discussion

Our study demonstrated that the variation in track abundance among species reflects different levels of forest ecological integrity. The Forest Landscape Integrity Index (FLII) effectively captures these variations and provides valuable insights into how different mammal species respond to varying levels of human disturbance in Finnish forest habitats. Preference for forests with higher FLII scores—indicating less human disturbance—tended to be more common for native and more sensitive mammal species, such as the forest reindeer, wolf, moose, wolverine, pine marten, lynx, and mountain hare. Conversely, more disturbed forests with lower FLII scores exhibit higher abundances of adaptable species that thrive in human-altered environments, including the brown hare, roe deer, red fox, and red squirrel.

This finding is further supported by the standardized coefficients presented in Supplementary Table S3, which shows large absolute effects of FLII on abundance in the models with FLII as the only forest-related variable. It seems that FLII alone serves as a strong indicator of forest integrity affecting species abundance. Notably, for two of the four species with negative associations with FLII, namely roe deer and brown hare, the FLII demonstrates a substantial effect size on track abundance. The brown hare, originally a steppe species from Central Asia and continental Europe, has expanded northward in Finland over the past three decades, partly due to its adaptability to various habitats, especially open fields (Thulin, 2023; Caravaggi et al. 2015). Similarly, the roe deer, recognized as the most widespread deer in Europe, adapts to diverse environments and relies on cultivated fields for foraging, especially in winter (Tinoco Torres et al. 2011).

Given FLII’s effectiveness in predicting the abundance of both forest dwelling species, and species adaptable to low-integrity habitats, it could be utilized to establish threshold values for conservation efforts. For instance, FLII could help forecast the expansion of species that thrive in human-impacted environments, such as the brown hare and roe deer.

However, it is important to note that incorporating forest cover and canopy cover as additional explanatory variables in the models, provides a more complete picture of the habitat preferences of some species, as evidenced by the substantial increases in their marginal R^2^. In particular, when forest cover and canopy cover were included as additional variables in the full model, the effects of FLII became less pronounced for many species (Supplementary Table S4). This suggests that while FLII is a valuable summarizing indicator of ecological integrity, the direct physical attributes of the habitat, such as the amount of forest cover and canopy cover, play a more immediate and significant role in determining species abundance.

Specifically in the study area, in fragmented forest landscapes, FLII may assign high scores to isolated forest patches naturally fragmented by undisturbed treeless peatlands or waterbodies. However, such small forest patches might not be optimal habitats for many species requiring large areas of continuous forest cover, or due to poor connectivity with other suitable habitat patches. Similarly, peatlands that are often classified as high-integrity forest areas according to FLII have sparse tree cover but are not suitable habitats for species that prefer dense canopy cover. Dense cover generally provides shelter and concealment, facilitating escape from predators (Thompson et al. 2003). For example, pine martens (Hargis and McCullough 1984) and weasels (Mougeot et al. 2020) use stands with dense cover to avoid avian predators. Canopy cover could also be an important environmental element for protection from humans, emphasizing the need for more nuanced habitat assessments.

## Conclusions

Our study supports the hypothesis that the Forest Landscape Integrity Index (FLII) provides a solid basis in measuring aspects of forest ecological integrity degradation on the biotitic community. However, integration with other environmental variables provides a more complete and accurate understanding of ecological dynamics.

Therefore, we recommend that future research focus on adapting these global indices to local contexts to achieve more accurate and meaningful assessments of forest health and biodiversity. Such adaptations will allow the development of more targeted conservation strategies that take into account regional forest variations and species-specific needs.

## Supplementary Information

Below is the link to the electronic supplementary material.

Supplementary file1 (DOCX 29 KB)

## Data Availability

Data on wildlife triangles that support the results of this study are available from the National Resources Institute Finland (LUKE), but restrictions from the Finnish government apply to the availability of these data, which were used under license for this study and are therefore not publicly available. However, the data are available upon reasonable request and with the permission of the National Resources Institute Finland (LUKE). The data on canopy cover and forest cover are available from https://kartta.luke.fi/opendata/valinta-en.html. The global raster on Forest Landscape Integrity Index (FLII) is available from https://www.forestintegrity.com/.

## Code availability

Code generated during the current study is available from the corresponding author on request.
