# Supplementary material for: Correlation between mammal track abundance and Forest Landscape Integrity Index validates actual forest ecological integrity
Source: Oecologia. 2024 Sep 4;206(1-2):61–72. doi: 10.1007/s00442-024-05613-z (PMC11489168; doi:10.1007/s00442-024-05613-z)
Supplement: Supplementary file 1 — Supplementary file1 (DOCX 29 KB) [file 442_2024_5613_MOESM1_ESM.docx]

**Snow track abundances**

**Article title:** Correlation between mammal track abundance and Forest Landscape Integrity Index validates actual forest ecological integrity.

**Journal name:** Oecologia

**Author names:** Francesca Malcangi, Andreas Lindén, Janne Sundell, John Loehr

**Affiliation and e-mail address of the corresponding author:** Lammi Biological Station, University of Helsinki. [francesca.malcangi@hesinki.fi](mailto:francesca.malcangi@hesinki.fi)

**Supplementary Table S1. Number of snow tracks and relative abundances for all triangles sampled across all years.**

| Species | N. tracks | Relative abundance % |
| --- | --- | --- |
| American mink | 824 | 0,38 |
| Brown Hare | 21998 | 10,06 |
| Eurasian lynx | 2620 | 1,20 |
| European otter | 1086 | 0,50 |
| Forest reindeer | 743 | 0,34 |
| Least weasel | 2168 | 0,99 |
| Moose | 25710 | 11,75 |
| Mountain Hare | 82836 | 37,87 |
| Pine Marten | 4500 | 2,06 |
| Raccoon Dog | 2877 | 1,32 |
| Red Fox | 25510 | 11,66 |
| Red Squirrel | 12578 | 5,75 |
| Roe Deer | 9173 | 4,19 |
| Stoat | 2343 | 1,07 |
| White-Tailed Deer | 22593 | 10,33 |
| Wolf | 324 | 0,15 |
| Wolverine | 879 | 0,40 |

**Supplementary Table S2. Number of snow tracks and relative abundances for all triangles sampled each year.**

| Species | 2016 | 2017 | 2018 | 2019 | 2020 |
| --- | --- | --- | --- | --- | --- |
| American mink | 0,39 | 0,34 | 0,32 | 0,35 | 0,52 |
| Brown Hare | 9,49 | 13,15 | 10,71 | 9,54 | 5,98 |
| Eurasian lynx | 1,31 | 1,23 | 1,21 | 0,86 | 1,36 |
| European otter | 0,44 | 0,56 | 0,64 | 0,34 | 0,45 |
| Forest reindeer | 0,38 | 0,27 | 0,35 | 0,51 | 0,15 |
| Least weasel | 1,17 | 0,81 | 1,02 | 0,79 | 1,20 |
| Moose | 12,18 | 11,89 | 13,19 | 11,43 | 9,31 |
| Mountain Hare | 37,17 | 34,51 | 30,76 | 41,57 | 49,34 |
| Pine Marten | 2,24 | 2,07 | 2,03 | 2,12 | 1,72 |
| Raccoon Dog | 1,23 | 1,85 | 0,69 | 1,68 | 1,07 |
| Red Fox | 11,30 | 12,34 | 12,54 | 11,40 | 10,28 |
| Red Squirrel | 5,01 | 5,81 | 4,75 | 5,86 | 8,03 |
| Roe Deer | 3,81 | 3,90 | 5,61 | 3,99 | 3,50 |
| Stoat | 1,69 | 0,85 | 0,75 | 0,84 | 1,18 |
| White-Tailed Deer | 11,76 | 9,96 | 14,89 | 8,30 | 4,85 |
| Wolf | 0,15 | 0,07 | 0,16 | 0,05 | 0,38 |
| Wolverine | 0,28 | 0,39 | 0,38 | 0,36 | 0,68 |

**Standardized coefficients (β) of the fixed effects predictors and their standard errors (SE) for FLII model (Table 3) and Full model (Table 4).**

Standardized coefficients **(β)** and their standard errors (SE) of the fixed effects predictors. These statistics were calculated by multiplying the original estimates with the sample standard deviations of the explanatory variables. The standardized coefficients describe the level of change in the linear predictor (response variable prediction on the log-scale) per SD of the explanatory variable. Therefore, they illustrate effect sizes, and explanatory abilities of the explanatory variables in relation to each other.

**Supplementary Table S3**

| Species | FLII | | Latitude | |
| --- | --- | --- | --- | --- |
|  | β | SE | β | SE |
| American mink | 0.204 | 0.117 | 0.439 | 0.166 |
| Brown Hare | -1.182 | 0.12 | -1.008 | 0.268 |
| Eurasian lynx | 0.273 | 0.094 | -0.336 | 0.194 |
| European otter | 0.104 | 0.103 | -0.019 | 0.148 |
| Forest reindeer | 1.385 | 0.478 | -0.219 | 0.835 |
| Least weasel | -0.096 | 0.103 | -0.029 | 0.103 |
| Moose | 0.581 | 0.082 | -0.414 | 0.113 |
| Mountain Hare | 0.244 | 0.066 | 0.085 | 0.121 |
| Pine Marten | 0.433 | 0.081 | -0.102 | 0.134 |
| Raccoon Dog | 0.03 | 0.101 | -1.498 | 0.158 |
| Red Fox | -0.133 | 0.065 | -0.209 | 0.131 |
| Red Squirrel | -0.165 | 0.073 | -0.348 | 0.077 |
| Roe Deer | -0.845 | 0.139 | -0.993 | 0.32 |
| Stoat | 0.007 | 0.094 | 0.578 | 0.091 |
| White-Tailed Deer | 0.122 | 0.149 | -3.557 | 0.463 |
| Wolf | 1.328 | 0.309 | -0.427 | 0.426 |
| Wolverine | 0.401 | 0.163 | 1.484 | 0.371 |

**Supplementary Table S4**

| Species | FLII | | Latitude | | Forest Cover | | Canopy Cover | |
| --- | --- | --- | --- | --- | --- | --- | --- | --- |
|  | β | SE | β | SE | β | SE | β | SE |
| American mink | 0.174 | 0.127 | 0.565 | 0.16 | 0.174 | 0.121 | 0.288 | 0.121 |
| Brown Hare | -0.422 | 0.122 | -0.902 | 0.2 | -1.057 | 0.086 | -0.039 | 0.124 |
| Eurasian Lynx | 0.181 | 0.1 | -0.268 | 0.189 | 0.296 | 0.096 | 0.281 | 0.1 |
| European otter | -0.07 | 0.113 | -0.14 | 0.111 | 0.385 | 0.097 | -0.089 | 0.086 |
| Forest reindeer | 1.532 | 0.54 | -0.228 | 0.858 | -0.236 | 0.378 | -0.032 | 0.528 |
| Least weasel | -0.075 | 0.112 | 0.067 | 0.109 | 0.004 | 0.088 | 0.235 | 0.087 |
| Moose | 0.168 | 0.083 | -0.653 | 0.109 | 0.984 | 0.076 | -0.199 | 0.071 |
| Mountain Hare | 0.089 | 0.071 | 0.185 | 0.091 | 0.405 | 0.059 | 0.246 | 0.063 |
| Pine Marten | 0.204 | 0.084 | -0.256 | 0.114 | 0.722 | 0.088 | -0.004 | 0.082 |
| Raccoon Dog | 0.16 | 0.107 | -1.342 | 0.163 | -0.277 | 0.085 | 0.305 | 0.113 |
| Red Fox | -0.044 | 0.07 | -0.298 | 0.124 | -0.227 | 0.056 | -0.267 | 0.065 |
| Red Squirrel | -0.071 | 0.079 | -0.253 | 0.081 | -0.165 | 0.061 | 0.187 | 0.064 |
| Roe Deer | -0.192 | 0.148 | -0.819 | 0.285 | -0.958 | 0.108 | 0.1 | 0.155 |
| Stoat | 0.1 | 0.102 | 0.737 | 0.1 | -0.11 | 0.081 | 0.328 | 0.079 |
| White-Tailed Deer | -0.063 | 0.167 | -3.654 | 0.468 | 0.298 | 0.121 | -0.182 | 0.196 |
| Wolf | 1.072 | 0.325 | -0.775 | 0.441 | 0.659 | 0.324 | -0.662 | 0.277 |
| Wolverine | 0.275 | 0.172 | 1.38 | 0.39 | 0.56 | 0.237 | -0.075 | 0.157 |
